# Long Non-Coding RNAs and the Innate Immune Response

**DOI:** 10.3390/ncrna5020034

**Published:** 2019-04-19

**Authors:** Marina R. Hadjicharalambous, Mark A. Lindsay

**Affiliations:** Department of Pharmacy and Pharmacology, University of Bath, Claverton Down, Bath, BA2 7AY, UK; mal37@bath.ac.uk

**Keywords:** long non-coding RNA, lncRNA, innate immunity, inflammation

## Abstract

Innate immunity provides the initial defence against infection and it is now clear that long non-coding RNAs (lncRNAs) are important regulators of this response. Following activation of the innate response, we commonly see rapid induction of these lncRNAs and this is often mediated via the pro-inflammatory transcription factor, nuclear factor-κB (NF-κB). Knockdown studies have shown that lncRNAs tend to act in trans to regulate the expression of multiple inflammatory mediators and other responses. Mechanistically, many lncRNAs have demonstrated acting through heterogeneous nuclear ribonucleoproteins, complexes that are implicated chromatin re-modelling, transcription process and translation. In addition, these lncRNAs have also been shown to interact with multiple other proteins involved in the regulation of chromatin re-modelling, as well as those proteins involved in intracellular immune signalling, which include NF-κB. In this review, we will describe the evidence that supports this emerging role of lncRNA in the innate immune response.

## 1. Introduction

Long non-coding RNAs (lncRNAs) are generally defined as endogenous cellular RNA molecules of more than 200 nucleotides in length that lack an open reading frame (ORF) of significant length (less than 100 amino acids) and contain 2 or more exons [1,2]. They were originally discovered in mice during large-scale sequencing of full-length cDNA libraries as part of the FANTOM project [3]. Currently there are 16,066 lncRNA genes and 29,566 lncRNA transcripts documented in the human genome database (version 29) of GENCODE, compared to 19,940 protein coding genes [4]. Accumulating sequencing data has meant that the number of lncRNAs continues to increase, including some that were previously mistakenly identified as protein coding genes [5].

As with protein-coding genes, the majority of lncRNAs appear to be transcribed by RNA polymerase II (RNAPII) [6], although there are a few exceptions that are transcribed by RNA polymerase III (RNAPIII) including the 7SL RNA genes [7]. LncRNAs have also been shown to be subjected to transcriptional editing such as splicing, polyadenylation and 5′ capping [6]. Subsequently, each lncRNA develops a final stable structure which shapes its unique cellular function, enabling it to interact with other molecules [8].

Despite the rapid increase in data relating to lncRNAs, little is known regarding their exact functions, mechanism of action or even how many different types of lncRNAs exist. Even though lncRNAs generally demonstrate poor evolutionary conservation [9], it is evident that they play important roles in multiple biological pathways, including modulation of the innate immune response [10,11].

## 2. Characteristics of lncRNAs

LncRNAs present several distinct characteristics when compared to mRNAs regarding their size, specificity, organisation and subcellular localisation. However, despite the differences, they also possess a lot of similarities to mRNAs regarding their biogenesis and form. 

## 3. LncRNAs and mRNAs Share Similar Biogenesis Pathways

LncRNAs are predominantly transcribed by RNAPII and most are spliced, polyadenylated at the 3′-end, 5′-end capped with 7-methylguanosine and are associated with similar histone markers to those of mRNAs [6,12,13]. Non-polyadenylated lncRNAs, including those associated with enhancer regions [14], are thought to be stabilised through other mechanisms. These include ribonuclease P (RNase P) cleavage to generate mature 3′-ends, the formation of circular molecular structures such as circRNAs or capping by small nucleolar ribonucleoproteins (snoRNP) complexes [15,16]. Unlike mRNAs which are known for their protein coding functions and translational potential, lncRNAs lack an ORF of significant length and quality [13] and are therefore deemed to have no translational capacity. However, interestingly, a study by Ruiz-Orera et al. suggested that lncRNAs may give rise to small novel peptides [17].

## 4. LncRNAs Are Expressed at Lower Levels Compared to mRNAs

LncRNAs are generally shorter in length, have fewer, but longer exons and are expressed at lower levels compared to mRNAs [13,18,19]. Indeed, lncRNAs demonstrated lower expression in all tissues compared to mRNAs except in the testes where they showed tissue-specific elevated expression levels [9,18,20,21]. These lower expression levels were initially advanced as evidence that lncRNAs were simply the result of transcriptional noise. This has since been disproved, following evidence showing biological functionality. 

## 5. LncRNAs Expression Is Cell and Tissue Specific

Transcriptome-wide studies demonstrated that expression of lncRNAs is specific for the cell, tissue, developmental or disease state, as well as highly dependent on context and time [13,18,20,22]. For example, Cabili et al. found that 78% of lincRNAs were tissue-specific across 24 tissue and cell types compared to approximately 19% of protein coding genes [18]. This specificity of lncRNA expression may explain their low levels of expression compared to mRNAs and indicates that they possess cell/tissue selective functions [23]. 

## 6. LncRNAs Show Poor Evolutionary Conservation 

Unlike protein coding genes, lncRNAs generally show poor evolutionary conservation [9,13,18] and this lack of sequence conservation has made it difficult to identify functional domains and to compare their biological significance across species [24]. In general, the exon regions of lincRNAs were shown to demonstrate higher conservation than random un-transcribed intragenic regions, although still considerably less than that observed in exon regions of protein coding genes. Interestingly, the conservation across the promoter regions of lncRNAs is comparable to that of protein coding genes [6,25]. This lack of conservation appears to be related to the rapid evolution of lincRNAs, most lincRNAs have no conserved orthologues [19]. In contrast to sequence conservation, thousands of lncRNAs were found to demonstrate highly conserved genomic positions (synteny) [26]. Despite the general observation of poor conservation, a small number of lncRNAs demonstrate high conservation at both the sequence and structure levels and include well-characterised lincRNAs such as *MALAT1* (metastasis associated lung adenocarcinoma transcript 1) and *NEAT1* (nuclear enriched abundant transcript 1) [27]. 

## 7. Subcellular Localisation of lncRNAs

After transcription in the nucleus, mRNA transcripts tend to be transported to the cytoplasm where they undergo translation. In contrast, lncRNAs are found both in the nucleus and the cytoplasm, although current evidence suggests that they are predominantly enriched in the former [13,28]. Nuclear lncRNAs include some of the best studied lncRNAs such as *NEAT1* [29], *MALAT1* [30] and *XIST* (X-inactive specific transcript) [31] where they are thought to regulate epigenetic modifications and mRNA processing. However, even though lncRNAs as a group are more enriched in the nucleus compared to mRNAs, cytoplasmic lncRNAs are reported to be expressed in higher numbers [22,32]. Interestingly, a report by van Heesch et al. [33] showed a 30% enrichment of lncRNAs in the cytoplasm and 38% in ribosomal fractions compared to just 17% in the nucleus. Additionally, ribosome-profiling experiments have found abundant numbers of lncRNAs associated with ribosomes, suggesting they may actually be translated [17,34]. However, further studies failed to detect protein products from the supposed translation of lncRNA ORFs, suggesting that ribosomes can distinguish between coding and non-coding transcripts, and concluding that lncRNAs are unlikely to encode peptides/proteins [35]. 

## 8. Classification of lncRNAs

**Figure 1:**
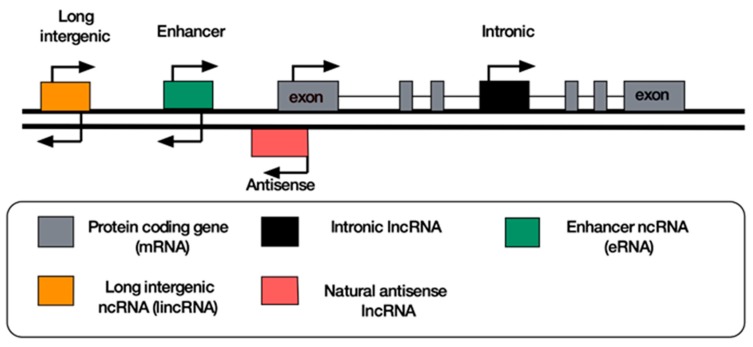
Classification of the most widely found lncRNAs according to their genomic location. Attempts to resolve the transcriptomic complexity of lncRNAs have led to their classification based on their genomic proximity to protein coding genes (mRNA). LincRNAs and eRNAs are stand-alone transcription units situated near protein coding genes. Intronic lncRNAs are found within the introns of protein coding genes, while antisense lncRNAs are transcribed from the opposite strand from the exonic regions of protein coding genes.

A convenient way to classify lncRNAs is based upon their position relative to well-established markers such as protein-coding genes (Figure 1). However, several lncRNAs do not fit into any of these categories as they present a combination of these qualities or they cover long genomic distances [36]. The most significant lncRNA classes are discussed below.

## 9. Antisense lncRNAs

Antisense lncRNAs, also known as natural antisense transcripts or NATs, are transcribed across the exons of protein-coding genes from the opposite strand, with varying degrees of overlap from partial to complete. Gene regulation by antisense transcripts occurs mainly in cis [37], where the antisense lncRNA interacts with its associated or neighbouring genes. GENCODE currently lists 5587 antisense lncRNA genes and 11,443 transcripts [4]. Interestingly, it is suggested that as much as 70% of protein coding genes have antisense counterparts [38,39]. 

## 10. Long Intergenic Non-Coding RNAs

Long intergenic non-coding RNAs (lincRNAs) are considered the largest and most significant group of lncRNAs, constituting approximately half the overall number of lncRNAs. GENCODE currently lists 7635 lincRNA genes giving rise to 14,379 lincRNA transcripts [4]. They are stand-alone transcripts that are located between protein coding genes and can regulate gene expression by acting either in cis or in trans. Prior to the advent of sequencing, lincRNAs were originally identified using two markers of active transcription: trimethylation of lysine 4 of histone H3 (H3K4me3) and trimethylation of lysine 36 of histone H3 (H3K36me3), present at their promoters during RNAPII transcription [6]. LincRNAs appear to have undergone rapid evolution and show variable conservation across species [21]. In a study by Ulitsky et al. mammalian lincRNA orthologues were found for just 5.1% of zebrafish lincRNA genes, demonstrating poor overall conservation when compared to protein coding genes [26]. Cabili et al. characterised the expression of human lincRNAs across 24 cell types and tissues using RNA-sequencing (RNA-seq). LincRNAs were found to have lower expression levels, fewer exons and to be expressed in a cell-specific manner compared to mRNAs (messenger RNA). LincRNA loci were typically found on average within 40kb of protein coding genes [18]. 

## 11. Enhancer RNAs

Enhancer RNA (eRNA) transcripts are found in both polyadenylated or non-polyadenylated forms and are reported to be bi-directionally expressed at active enhancer regions of the genome [14]. Enhancers are genomic regions located near protein coding genes which contribute to the initiation of transcription by promoting the binding of transcription factors (TFs) and other co-factors. Notably, a study by Kim et al. [40] revealed RNAPII-mediated transcription of eRNAs from enhancer regions in the presence of histone H3 monomethylated at lysine 4 (H3K4me1) which correlated with the activity of mRNA synthesis. As such, eRNAs are mainly thought to be *cis*-acting lncRNAs which control promoter and enhancer interactions as well as chromatin structures; resulting in the regulation of gene expression by promoting transcription of neighbouring genes [41,42]. Hence, eRNAs synthesis and enhancer activity are thought to be strongly correlated in regulating the transcriptional activity of neighbouring genes. 

## 12. Intronic RNAs

Intronic lncRNAs are entirely transcribed from the introns of annotated protein coding genes in either a sense or antisense direction. These lncRNAs have been associated with the nesting of small ncRNAs such as microRNAs (miRNAs) and small nucleolar RNAs (snoRNAs) as well as circular non-coding RNAs (circRNAs) [43]. In a study by Ayupe et al. intronic RNAs showed evidence of RNAPII-mediated transcription and 5′-cap modifications [44]. The functions of intronic lncRNAs remain largely unclear as they are a relatively unexplored class of lncRNAs and further investigation into their mechanisms of regulation is necessary. However, it has been suggested that intronic lncRNAs are often co-transcribed with their host protein coding gene, thus possibly sharing strong regulatory features and relationships with their host gene [45]. 

## 13. circRNAs

circRNAs are a class of recently discovered regulatory RNAs which were found to interact and regulate the activity of miRNAs, hence usually referred to as “miRNA sponges” [46,47]. A study by Memczak et al. identified approximately 2000 human, 1900 mouse and 700 nematode circRNAs that may be expressed from both coding and non-coding genomic loci [15]. circRNAs have been found to localise primarily in the cytoplasm and inhibit miRNAs by acting as miRNA-competing transcripts [48,49]. Interestingly, another type of circRNAs, known as circular intronic RNAs (ciRNAs) were shown to be located in the nucleus, acting as *cis*-regulators of RNAPII-mediated transcription and expression of parent genes [43].

## 14. Pseudogenes

The non-coding genome also gives rise to pseudogenes which are derived from protein coding genes that lose their coding potential through evolution [50]. There are currently 14,729 pseudogenes annotated in GENCODE (version 29). However, it appears that a number of pseudogenes may potentially regulate the expression of protein coding genes by processing into short interfering RNAs or acting as miRNA decoys (sponges) to regulate oncogenes during cancer progression [51]. Pseudogenes were also reported to be co-transcribed with their parent gene, functioning as antisense transcripts or even producing short peptides [52]. 

## 15. LncRNAs and the Regulation of Biological Function

**Figure 2:**
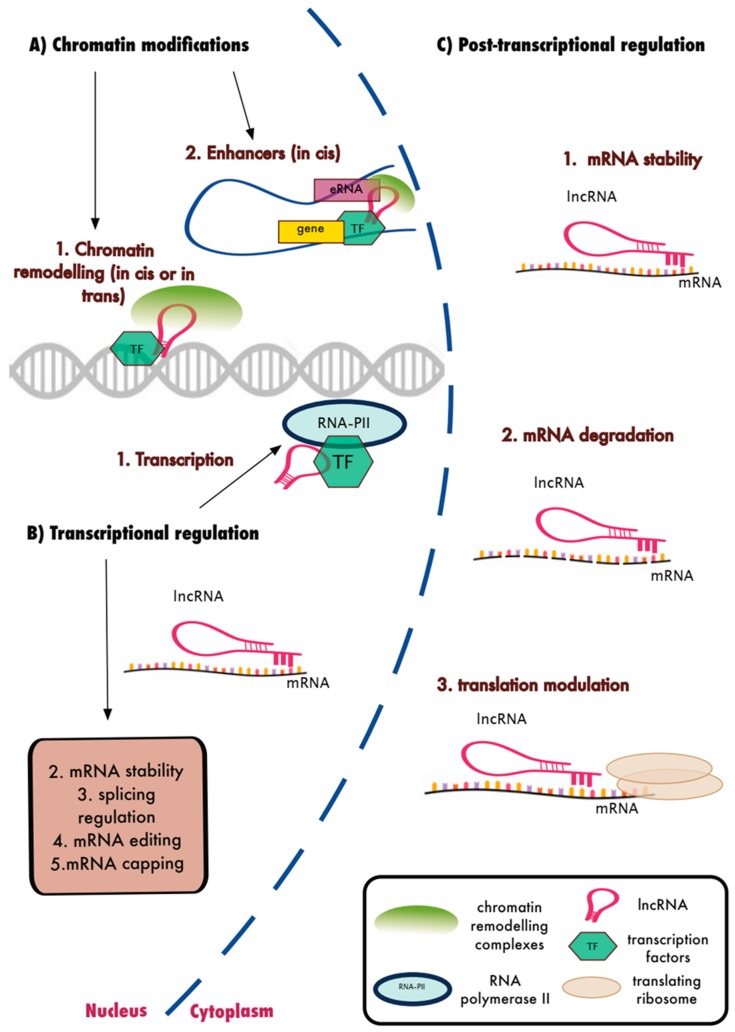
Biological functions of lncRNAs. LncRNAs were shown to interact with protein coding genes and their transcripts to regulate gene expression. (**A**) Nuclear lncRNAs interact with chromatin remodelling factors and TFs to regulate the expression of neighbouring or distal genes. (**B**) Nuclear lncRNAs also regulate transcription and several other transcriptional events of RNA processing. (**C**) Cytoplasmic lncRNAs were shown to interfere with post-transcriptional regulation such as mRNA stability and degradation as well as translational regulation of mRNAs.

LncRNAs are a relatively recently identified class of RNA molecules, and hence their functions and mechanism of action remain largely unexplored. They were originally considered transcriptional *noise* and although the majority are still likely to be biologically inactive, there is emerging evidence that a substantial number have functional activity. In particular, a number of highly expressed lncRNAs such as *HOTAIR* (HOX transcript antisense RNA) [53], *XIST* [31,54], *MALAT1* [30], and *H19* [55] have been studied in more depth and are relatively well-characterised. Nuclear located lncRNAs are generally associated with chromatin modifications, transcriptional regulation and RNA processing, whereas cytoplasmic lncRNAs have been linked with mRNA stability/translation and as direct agonists/antagonists of protein expression (Figure 2). As such, lncRNA transcripts have been associated with the regulation of all aspects of mRNA processing and protein activity [56]. 

When attempting to examine the role of a lncRNA in a biological response such as innate immunity, it is common to begin by examining their profile of lncRNA expression using techniques such as high throughput RNA sequencing and microarrays. Given the large numbers of lncRNAs identified, experimenters will then often focus upon those that are differentially or selectively expressed, and which are present at high levels. Subsequent functional studies are often undertaken using various RNA silencing techniques both in vitro and in vivo, and in case of the innate immune response have included antisense locked nucleic acid (LNA) [57,58,59,60], small interfering RNAs (siRNAs) [61,62,63] and the CRISPR/Cas9 system [64,65]. Pharmacological inhibitors and gene knockdown approaches can then be employed to examine the intracellular signaling pathways and transcription factors that regulate lncRNA expression. Arguably the biggest challenge is determining their mechanism of action which will commonly begin by investigation of the lncRNA-protein and lncRNA-RNA/DNA interactions using various pull-down techniques such as RNA immunoprecipitation (RIP) [63], capture hybridization analysis of RNA targets (CHART) and chromatin isolation by RNA purification (ChIRP) [66]. 

## 16. LncRNAs and the Innate Immune Response

The innate immune system is the first line of host defence against infection and involves the recognition and elimination of pathogens. The initial recognition of common pathogenic components including pathogen-associated molecular patterns (PAMPs) and damage-associated molecular patterns (DAMPs) is mediated via pattern recognition receptors (PRRs) such as toll-like receptors (TLRs), the nucleotide binding and oligomerisation domain (NOD) like receptors (NLRs), RIG-I-like receptors (RLRs) and C-type lectin receptors (CLRs). This leads to the subsequent activation of the inflammasome and multiple transcription factors including nuclear factor-κB (NF-κB) and the interferon response factors (IRFs), that mediate the inflammatory response involved in the removal of pathogens. These receptors are expressed by the myeloid cells associated with the innate immune system including monocytes, macrophages and dendritic cells, as well as tissue associated cells such as the epithelium and fibroblasts [67]. LncRNA are now implicated in multiple aspects of the innate immune response including the maintenance of hematopoietic stems cells, the differentiation and apoptosis of myeloid cell and the activation of monocytes, macrophages and dendritic cells. An overview of the function and mechanism of action of ncRNAs in the innate immune response is given in Table 1 and is described in more detail in the following sections.

## 17. LncRNAs in Hematopoietic Development, Differentiation and Apoptosis

The maturation of hematopoietic stem cells (HSCs) is a critical step in the development of other blood cells which are grouped into either the lymphoid or the myeloid lineage. Cells of the lymphoid lineage include B and T cells as well as natural killer (NK) cells, while myeloid cells include granulocytes, monocytes, macrophages and megakaryocytes [68]. 

A study by Venkatraman et al. has shown that the lncRNA *H19* plays a crucial role in maintaining adult haematopoietic stem cell quiescence [69]. The maternally specific upstream region of *H19*, named H19-DMR, activates the Igf2–Igfr1 pathway by the translocation of the phosphorylated FoxO3 (Forkhead box O3) to the cytoplasm when inactivated. This leads to enhanced *Igf2* expression and Igfr1 translation which play a significant role in the elevated activation and proliferation of HSCs. 

A different study, identified 159 novel lncRNAs enriched in purified mouse HSCs (LncHSCs) using deep sequencing technology [70]. Further analysis of the nucleus-enriched lncRNAs *LncHSC-1* and *LncHSC-2***,** demonstrated differential expression between wild-type and Dnmt3a (DNA Methyltransferase 3 Alpha) knock-out HSCs. Significantly, *LncHSC-1* was found to be involved in myeloid differentiation while *LncHSC-2* demonstrated a role in T cell differentiation and HSC restoration. 

The lncRNA *Morrbid* (myeloid RNA regulator of Bim-induced death) was found to regulate the lifespan of short-lived cells such as monocytes, as well as neutrophils and eosinophils, which make up the majority of human white blood cells. Evidence shows that Morrbid controls the survival of these cells by regulating the transcription of *Bcl2l11*, a proapoptotic neighboring gene also known as *Bim*, through the enrichment of the Polycomb Repressive Complex PRC2 at its promoter to promote trimethylation of histone H3 at lysine 27 (H3K27me3) modifications [64]. 

## 18. LncRNAs in Monocytes and Macrophage Activation

Pathogen mediated activation of myeloid cells such as monocytes, macrophages and dendritic leads to the rapid release of inflammatory mediators such as cytokine and chemokines, as well as multiple other response that are associated with inflammation including adhesion, chemotaxis, phagocytosis, NADPD oxidase activation, antigen presentation and monocyte to macrophage differentiation [71]. To date, the majority of mechanistic and functional studies have examined the production of inflammatory mediator in response to lipopolysaccharide (LPS), a component of the wall of gram—ve bacteria, which acts predominantly via the TLR4 receptor. These studies have identified a large number of functional lncRNAs, in both humans and mice, and to aid in this description these will be divided to antisense and lincRNAs.

## 19. Antisense lncRNAs in Monocytes and Macrophage Activation

Cyclooxygenase-2 (COX-2), also known as prostaglandin-endoperoxide synthase (PTGS), is an inducible enzyme that is involved in the formation of inflammatory prostanoids. The importance of the enzyme is demonstrated by the fact that pharmaceutical inhibition of COX-2 attenuates both inflammation and pain in patients. Interestingly, a number of lncRNAs have been shown to regulate COX-2 expression including *lincRNA-COX2* (see lincRNA section) and the p50-associated COX-2 extragenic RNA (*PACER*) [61]. *PACER* is located upstream and is transcribed in the antisense direction through the COX-2 (Cyclooxygenase-2) promoter in response to LPS and PMA (phorbol 12-myristate 13-acetate). It was shown to induce the expression of the *COX-2* gene in human U937 monocytic cells by interacting directly with the inhibitory NF-κB (nuclear factor-κB) p50 homodimer. Thus, by occluding the repressive NF-κΒ p50 complex in human monocytes and differentiated macrophages, this enables the binding of the active p65/RelA NF-κΒ complex and the subsequent recruitment of histone acetyltransferase (HAT) p300, that leads to increased histone acetylation and assembly of RNA-PII pre-initiation complexes [61]. 

A number of other antisense lncRNAs have also been shown to be induced in response to LPS, including *lnc-IL7R* (interleukin-7 receptor α-subunit) in human THP-1 monocytes [72], *NRIR* (Negative Regulator of the IFN Response) in human monocytes from systemic sclerosis patients [73], *Ptprj-as1* [74] and *Mirt2* [75] in mouse macrophages, as well as *IL7-AS* (antisense to the IL7 gene promoter) in both human THP-1 monocytes and mouse RAW 264.7 macrophages [59]. In addition to LPS, *AS-IL1α* was shown to be induced upon infection with *Listeria monocytogenes* in the spleens of C57BL/6 mice and in isolated BMDMs. In contrast, expression of the sense transcript *lnc-13* (which overlaps with Il18rap) was reduced following LPS exposure in primary human and mouse macrophages [76]. Despite being antisense transcripts, knockdown studies have showed that *lnc-IL7R* [72], *NRIR* [73], *Mirt2* [75] and *IL7-AS* [59] regulate the expression of multiple inflammatory mediators by acting in trans. *Mirt2* was also found to regulate macrophage polarization [75]. The similar pattern of expression levels of the *Ptprj* gene and its *Ptprj-as1* antisense indicates that this lncRNA co-regulates its associated gene by acting in cis [74]. Examination of the pathways regulating their induction indicated that *NRIR* [73] and *IL7-AS* [59] expression is mediated via type I IFN (interferon) and NF-κB pathways, respectively. Mechanistic studies have shown that *Mirt2* decreases expression of inflammatory cytokines by attenuating Lys63-ubiquitination and oligomerisation of TRAF6 and thereby inhibiting the activation of the NF-κB and MAPK (mitogen-activated protein kinase) pathways. *Lnc-13* is thought to regulate gene expression by interacting directly with hnRNPL (heterogenous nuclear ribonucleoprotein L) to mediate the localisation of Hdac1 (Histone deacetylase 1) on chromatin [76]. In contrast, *AS-IL1α* which is encoded within the IL-1α locus, was found to regulate *IL-1α* by recruiting RNAP- II to the IL-1α promoter during transcription induced by LPS [77]. 

## 20. *LincRNAs* in Monocytes and Macrophage Activation

As with antisense lncRNAs, the naming of lincRNAs is often based upon the nearest protein coding gene. Like PACER, *lincRNA-COX2* is located proximal to the *COX2 (Ptgs2)* gene and was originally shown by Carpenter et al. [78] to regulate the expression of multiple genes in non-stimulated mouse bone marrow derived macrophages (BMDMs). In addition, following exposure to the TLR1/2 agonist Pam3CSK_4_ (palmitoyl-3-cysteinyl-seryl-(lysyl)_4_), *lincRNA-COX2* induction which is mediated via NF-κB, was demonstrated to regulate the downregulation and upregulate of 787 and 713 genes, respectively [78]. Mechanistic studies identified hnRNP-A/B and hnRNP-A2/B1 (heterogeneous nuclear ribonucleoproteins) as potential binding partners of *lincRNA-COX2*. Subsequent reports have demonstrated that *linc-COX2* also regulates the late inflammatory response upon exposure to LPS in murine macrophages [79]. In this paper, *lincRNA-COX2* was shown to be integrated into the SWI/SNF (Switch/Sucrose NonFermentable) complex that controls the activity of NF-κΒ and the chromatin re-modelling associated with the transcription of the late-primary inflammatory genes. Another study by Xue et al. [80] has identified *lincRNA-COX2* as a modulator of the inflammasome sensor NLRP3 and ASC adaptor, by binding to NF-κΒ p65 upon LPS stimulation of BMDMs. *LincRNA-COX2* knockdown was found to inhibit caspase-1 activation, decrease secretion of IL-1β and TRIF (TIR-domain-containing adapter-inducing interferon-β) cleavage; ultimately increasing TRIF-mediated BMDM autophagy. In support of these earlier studies, Elling et al. [81] employed a knockout mouse model to confirm that *lincRNA-COX2* regulated the inflammatory gene expression in trans during LPS-induced sepsis. Interestingly, the study revealed that *lincRNA-COX2* might act as an eRNA in cis since it was observed that knockout inhibited the expression of neighboring genes across a number of different tissues. 

The conserved lncRNA *MALAT1* is another lincRNA that has been reported to regulate the innate immune response in multiple publications. A study by Zhao et al. [82] demonstrated that *MALAT1* was upregulated in response to LPS activation and knock-down studies showed increased expression of inflammatory cytokines such as TNF-α, and IL-6 but had no effect on IL-1β. *MALAT1* was also shown to inhibit the activity of NF-κB by binding to the nuclear p65/p50 heterodimer; thus regulating the activity of other NF-κB dependent genes. A more recent study by Cui et al. [83] demonstrated that *MALAT1* regulates macrophage activation in response to pulmonary injury. *MALAT1* expression was found to be elevated in LPS-activated macrophages and downregulated in IL-4-exposed cells. Interestingly, knock-down of *MALAT1* increased IL-4-induced differentiation of M2 macrophages and induced a pro-fibrotic cell phenotype. *MALAT1* knock-out mice also demonstrated decreased pulmonary inflammation accompanied with increased lung fibrosis. 

Gene profiling has also shown differential expression of multiple other lincRNAs in response to inflammatory mediators such as LPS, including *THRIL* (TNF-α and hnRNPL related immunoregulatory lincRNA) [84] and *IL1β-RBT46* [85] in human, as well as *lincRNA-EPS* (erythroid prosurvival or Ttc39aos1) [86], *linc-Tnfaip3* (tumor necrosis factor α-induced protein 3) [87], *NTT* (noncoding transcript in T cells) [88] and *FIRRE* [89] in mice (Table 1). *lincRNA-EPS* was originally discovered as a regulator of differentiation and apoptosis in erythroid cells [90] whilst *NTT* (noncoding transcript in T cells) was shown to be elevated in peripheral blood mononuclear cells (PBMCs) from rheumatoid arthritis (RA) patients [88]. Investigation of the mechanisms that drive lincRNA transcription indicated that *IL1β-RBT46* [85] and *FIRRE* [89] expression was mediated via NF-κB, whilst *NTT* is modulated by C/EBPβ [88].

Knockdown studies have demonstrated that these lincRNAs also regulate the expression of multiple inflammatory genes in trans. In a similar fashion to *lincRNA-COX2* and *Lnc-13*, the actions of many of these lincRNAs has been linked to an interaction with heterogeneous nuclear ribonucleoproteins (hnRNPs). Thus, *THRIL* [84] and *lincRNA-EPS* [91] were shown to interact with hnRNPL, whilst in-depth analysis in *lincRNA-EPS* deficient mice demonstrated this to be linked to elevated levels of H3K4me3, alternating nucleosome positioning and chromatin accessibility at the promoters of immune responsive genes [91]. *FIRRE* was found to interact with hnRNP-U to regulate the stability of inflammatory-associated genes by targeting the adenylate-uridylate–rich elements (AREs) of mRNAs [89]. Similarly, *NTT* (noncoding transcript in T cells) was demonstrated to bind the promoter regions of the *PBOV1* (prostate and breast cancer overexpressed 1) gene via hnRNP-U. Elevated *PBOV1* then resulted in expression of the *IL-10* and *CXCL10* mRNA levels, macrophage differentiation and cell cycle G1 arrest. Of relevance, this C/EBPβ/NTT/PBOV1 complex is suggested to play a regulatory role in the inflammatory response of monocytes associated with pathogenesis of rheumatoid arthritis [88]. In contrast to an action mediated via hnRNPs, *linc-Tnfaip3* was shown to regulate the activity of inflammatory genes by controlling the assembly of the NF-κB/Hmgb1 (high-mobility group box 1) complex upon LPS stimulation. 

Finally, Covarrubias et al. [65] used the CRISPR/Cas9 gene deletion technology to study lncRNAs that regulate the NF-κB signalling pathway and demonstrated that *lincRNA-Cox2* and *lincRNA-AK170409* are crucial regulators of inflammation. As such, the nuclear-enriched *lincRNA-AK170409* was found to be a novel modulator of several genes associated with inflammation and *lincRNA-Cox2* was shown to mediate IκBα degradation in the cytoplasm of LPS-stimulated immortalized murine bone marrow–derived macrophages (iBMDM). 

## 21. LncRNAs in Dendritic Cells

DCs are specialised antigen-presenting cells (APCs) that act as a link between innate and adaptive immunity [92]. Indeed, the first report examining the potential role of lncRNAs in the immune response involved studies of LPS-induced activation of CD11C^+^ mouse bone marrow-derived DCs. This report by Guttman et al. [6] employed chromatin markers such as H3K4me3 and H3K26me3 to demonstrates upregulation of 20 lincRNAs including *lincRNA-COX2*. 

**Figure 3:**
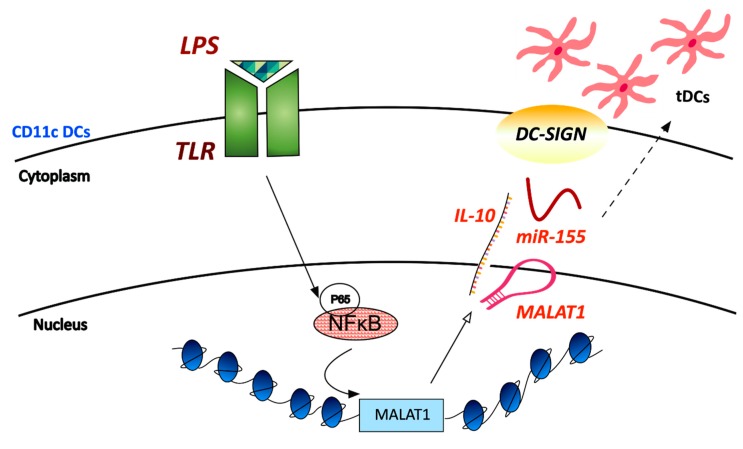
Expression of *MALAT1* is increased in response to inflammatory stimuli in CD11c^+^ cells of tolerized allograft cardiac tissue. LPS-induced transcription of *MALAT1* was shown to be dependent on the p65- associated activation of the NF-κB pathway and to increase secretion of the immunosuppressive cytokine IL-10. DC-SIGN (dendritic cell- specific intercellular adhesion molecule-3 grabbing nonintegrin) was also shown to be controlled by *MALAT1* and to act as a miR-155 sponge to maintain the tolerogenic ability of the tDCs (tolerogenic DCs).

Subsequent studies have found *MALAT1* to be upregulated via an NF-κB-dependent, in CD11c^+^ DCs from LPS-stimulated mice [93]. Overexpression of *MALAT1* caused a more tolerogenic phenotype in LPS-activated DCs and elevated IL-10 production. Evidence suggests that tolerogenic DCs build immune tolerance by regulating T cell activity and generating immunosuppressive regulatory T cells (Tregs). Thus, Wu et al. [93] presents lncRNA *MALAT1* as a novel modulator of tolerogenic DCs and immune tolerance by regulating the activity of the miRNA-155/DC-SIGN (dendritic cell- specific intercellular adhesion molecule-3 grabbing nonintegrin)/IL10 axis (Figure 3). 

The *lnc-DC* was also shown to be expressed in human conventional DCs and to regulate cell differentiation by interacting directly with STAT3 (signal transducer and activator of transcription 3). More specifically, knockdown studies have shown that *lnc-DC* prevented differentiation of BMDMs in vivo and human monocytes in vitro. *Lnc-DC* was found to directly bind to STA3 in the cytoplasm to promote phosphorylation of STAT3 on Y705 (tyrosine-705), and to prevent interaction and dephosphorylation by the tyrosine-specific protein phosphatase SHP1 [94]. 

## 22. LncRNAs in Fibroblasts

Although fibroblasts are not considered to be in the group of immune cells, they have a crucial role in wound healing and are important in providing a physical barrier as a line of defence to pathogens. Fibroblasts produce inflammatory mediators in response to trauma or infection and they may express TLRs, synthesise antimicrobial peptides and trigger the innate immune response [95]. 

**Figure 4:**
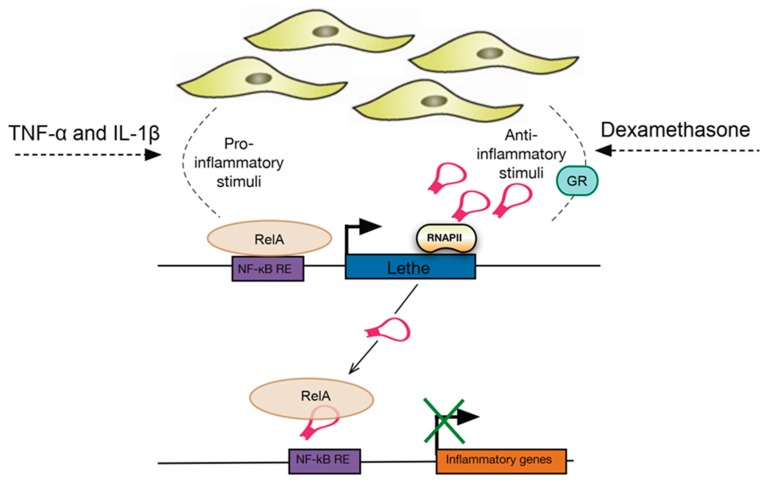
*Lethe* regulates gene expression upon exposure to TNF-α, IL-1β and dexamethasone in fibroblasts. Activation of *Lethe* by either pro- or anti-inflammatory stimuli was found to inhibit expression of inflammatory genes by binding directly to RelA and preventing NF-κB binding to DNA. Therefore, *Lethe* is suggested to act as a negative regulator of NF-κB and the activation of the inflammatory response. GR, glucocorticoid receptor; NF-κB RE, NF-κB response element; RelA, transcription factor p65.

The lncRNA *Lethe,* transcribed from the *Rps15a* pseudogene, was the first lncRNA found to play a role in inflammation in mouse embryonic fibroblasts. The study by Rapicavoli et al. [96] characterised *Lethe* as a 697bp long unspliced RNA located on chromosome 4 which is directly regulated by TNF-α, IL-1β and dexamethasone. RNA-seq analysis identified several lncRNAs that were differentially expressed upon TNF-α stimulation including 54 pseudogenes, of which a significant number were shown to be regulated by NF-κB. More specifically, further work focused on the role of *Lethe* in inflammation revealed that it is expressed in response to both TNF-α and IL-1β cytokines and it is primarily associated with chromatin. Interestingly, *Lethe* was shown to inhibit NF-κB DNA binding activity by directly interacting with the NF-κB subunit RelA, acting as a negative regulator of the inflammatory response (Figure 4). A more recent study by Zgheib et al. [97] investigated the role of *Lethe* in mouse macrophages to show that *Lethe* regulates ROS (reactive oxygen species) production in a model of diabetic wound healing by modulating NF-κB signalling and the expression of the *NOX2* (NADPH oxidase 2) gene.

Interestingly, *IL7AS* and *MIR3142HG* were also found to be implicated in the inflammatory response of primary human lung fibroblasts in response to IL-1β activation [60]. RNA-seq analysis identified 14 lncRNAs to be differentially expressed upon stimulation, including *IL7AS* and *MIR3142HG* which demonstrated the most significant upregulation. Knockdown studies showed the *IL7AS* was a negative regulator of IL-6 release, while *MIR3142HG* was a positive regulator of CCL2 and IL-8 protein release and both lncRNAs were found to be regulated by the NF-κB pathway. 

## 23. LncRNAs in Epithelial Cells

Similar to fibroblasts, epithelial cells are not considered to be primarily immune cells; however, they play a crucial role in the initiation, regulation and maintenance of the innate immune response. Moreover, epithelial cells may trigger the adaptive immune response as well as inhibit potential excessive immune stimuli to prevent damage to the epithelium [98]. 

IL-1β was shown to drive the expression of lncRNAs in human epithelial cells. More specifically, 34 lncRNAs were differentially expressed in response to IL-1β activation including *IL7AS* which was amongst the most significantly upregulated lncRNAs. *IL7AS* was found to mediate the inflammatory activity of the A549 cell line by regulating the expression of IL-6 mRNA and protein release upon knockdown studies [59]. 

*LincRNA-COX2* was found to also modulate inflammation in murine intestinal epithelial cells [99]. *LincRNA-COX2* was shown to be one of the most significantly upregulated genes in response to TNF-α stimulation and its induced transcription was dependent upon the activation of the NF-κB signalling pathway. Silencing of *lincRNA-COX2* increased the expression of the *Il12b* gene by recruiting the Mi-2/NuRD (Mi-2/nucleosome remodelling and deacetylase) repressor complex to its promoter region as well as by promoting histone modifications at the Il12b promoter upon TNF-α activation.

## 24. LncRNAs and Viral Infections 

The nuclear located 4-kb lncRNA *NEAT1* (nuclear paraspeckle assembly transcript 1) was originally shown to be essential for the structure and formation of paraspeckles [29]. However, its expression and the increased formation of paraspeckles has also been associated with viral infections such as influenza and herpes simplex virus-1 (HSV-1), as well activation following exposure to the immunostimulant polyinosinic:polycytidylic acid (poly I:C). NEAT1 was demonstrated to induce the expression of genes associated with viral infections such as *IL8*. Significantly, the *NEAT1* binding splicing factor proline/glutamine-rich (SFPQ) appeared to repress *IL8* expression; thus, *NEAT1* was found to relocate SFPQ to the paraspeckles in order to remove SFPQ from the IL-8 promoter and initiate transcription of *IL8* [100]. A different study by Morchikh et al. [101], identified a ribonuclear complex named HDP-RNP around the lncRNA *NEAT1* and *HEXIM1* to control the DNA-mediated innate immune response by regulating the cGAS (cyclic GMP-AMP synthase)-STING (stimulator of interferon genes) pathway. Imamura et al. [102] has also demonstrated that *NEAT* has a role in bacterial infection following studies of the nuclear lncRNAs *Salmonella* infected HeLa cells. Interestingly, inhibition of the lncRNAs *NEAT1v2* and *eRNA07573* resulted in elevated susceptibility to the bacterium and altered-regulation of genes associated with the innate immune response. In contrast to uninfected cells, *Salmonella*-exposed cells showed decreased levels of the nuclear exosome targeting (NEXT) complex, RRP6 (EXOSC10) and the RNA helicase MTR4, leading to the stabilisation of unstable RNAs; suggesting a potential novel role of these RNAs to the anti-bacterial cellular response. 

The cytoplasmic IFN-induced lncRNA, *lnc-Lsm3b*, was also recently found to interact with viral RNA molecules to inactivate the well-studied PRR, RIG-I (retinoic acid-inducible gene-I) function towards the later stages of the innate response [63]. Structural motifs of this lncRNA were found to be essential for optimal RIG-I protein binding and the modulation of IFN type I production to regulate the innate response and maintain immune homeostasis. 

The lncRNA *ITPRIP-1* (*lncITPRIP-1*) was also shown to play a significant role in regulating innate immunity in response to viral infection. According to recent evidence by Xie et al. [103], *lncITPRIP-1* interacts with the C-terminus of the melanoma differentiation-associated gene 5 (*MDA5*) to regulate the IFN signalling response. The study also demonstrated that hepatitis C virus (HCV) replication may be mediated by *MDA5* and its interaction with *lncITPRIP-1*. *MDA5* and *lncITPRIP-1* expression levels were correlated, indicating a co-dependent working mechanism to regulate the innate immune response during viral invasion. 

## 25. Future Perspectives 

It is now clear that lncRNAs are central regulators of the production of inflammatory mediators associated with the activation of the innate immune response. However, little is still known regarding their role in myeloid cell proliferation/differentiation or other responses involved in pathogen clearance, including adhesion, chemotaxis, exocytosis, phagocytosis and antigen presentation. Furthermore, despite the increasing number of proteins that have been shown to mediated the actions of lncRNAs, little is known about the domains that are responsible for these lncRNA:protein interactions. This is important because it would permit the identification of functional lncRNA based on sequence and/or structure. To date, this process has been hindered by their cell/tissue specific expression and poor evolutionary conservation, with the majority being expressed exclusively in either human or mouse. However, there are a number of exceptions to this rule include *IL7-AS* [59], *lnc-13* [76], *MALAT1* [82,83] and *FIRRE* [89], that have been shown to be expressed and regulate the inflammatory response in both species, and detailed mechanistic analysis of these specific lncRNA could begin to reveal these domains. Finally, changes in lncRNA expression and the presence of polymorphisms are increasingly implicated in the development of disease, an observation that could make lncRNA novel drug targets for conditions such as autoimmune diseases, allergies, chronic inflammation and infection. 

## Figures and Tables

**Table 1:** LncRNAs associated with the innate immune response.

LncRNA Name	Stimuli	Cell Type	Function	Mechanism	Refs
H19	N/A	Mouse hematopoietic stem cells	Maintains HSC quiescence	Regulates the Igf2-Igfr1 pathway via the translocation of FOX3 to the cytoplasm	[69]
LncHSC-1/2	N/A	Mouse hematopoietic stem cells	LncHSC-1 regulates myeloid differentiation and LncHSC-2 cell self-renewal and differentiation	LncHSC-2 recruits the hematopoietic TF E2A to its binding sites	[70]
PACER	PMA and LPS	Human monocytes (U937)	Promotes COX2 expression	Binds to the repressive p50 NF-κΒ subunit of COX2 promoter to enable p300 HAT recruitment in order to increase histone acetylation and initiate the assembly of RNAP-II complexes	[61]
Morrbid	N/A	Human and mouse monocytes, neutrophils, eosinophils	Controls the lifespan of monocytes, neutrophils and eosinophils	Regulates Bcl2l11 (Bim) transcription by promoting PCR2 enrichment at its promoter and deposition of H3K27me3	[64]
THRIL	PMA Pam3CSK_4_	Human monocytes (THP-1)	Regulates the expression of the innate-associated mediators TNF-α, CCL1, IL-8, CSF1 and CXC10	Forms a functional lncRNA-hnRNPL complex in order to regulate TNF-α transcription by binding to its promoter	[84]
LincRNA-COX2	Pam3CSK_4_	Mouse bone marrow derived macrophages	Regulates the expression of several immune genes	Interacts with the nuclear proteins hnRNP-A/B and hnRNP-A2/B1	[6,78,79,80,81,99]
	LPS		Regulates the NLRP3 inflammasome sensor and ASC adaptor as well as autophagy	Binds to p65 NF-κΒ subunit to promote its transcription. It was also found to regulate TRIF-mediated autophagy via caspase-1 activation	
	Multiple	LincRNA-Cox2 deficient mice and macrophages	Regulates the expression of several immune genes	Functions as an eRNA to regulate the activity of the* COX2* gene but also demonstrates in trans-regulation of immune-associated genes in vivo	
	LPS	Mouse macrophages (RAW 264.7 and primary peritoneal)	Regulates the expression of NF-κΒ-regulated inflammatory genes	Interacts with the SWI/SNF complex to regulate the assembly of NF-κΒ subunits and chromatin remodelling	
	TLR4 ligand	Mouse bone-marrow-derived dendritic cells	N/A	Demonstrates NF-κΒ-dependent expression	
	TNF-α	Murine intestinal epithelial cells (IEC4.1 cell line)	Regulates the expression of the *Il12b *gene	Demonstrates NF-κΒ-dependent expression and promotes the recruitment of the Mi-2/NuRD complex to the *Il12b* promoter	
AS-IL1α	TLR ligands and *Listeria monocytogenes*	Mouse bone marrow derived macrophages	Regulates IL-1α transcription	Facilitates RNAP-II recruitment to the IL-1α locus and demonstrates NF-κΒ-dependent expression	[77]
IL1β-eRNA IL1β-RBT46	LPS	Human monocytes (THP-1 and primary)	Regulate the expression of IL-1β and CXCL8	Demonstrate NF-κΒ-dependent expression	[85]
Lnc-IL7R	LPS	Human monocytes (THP-1)	Regulates the expression of the inflammatory mediators IL-6, IL-8, E-selectin and VCAM-1	Regulates deposition of H3K27me3 at the promoters of the E-selectin and VCAM-1 genes	[72]
Ptprj-as1	LPS Pam3Cys	Mouse bone marrow derived macrophages and RAW 264.7	N/A	N/A	[74]
IL7AS	LPS	Human monocytes (THP-1), mouse macrophages (RAW 264.7)	Regulates IL-6 expression and release	Demonstrates NF-κΒ-dependent expression	[59,60]
	IL-1β	Human epithelial cells (A549 cell line) Human lung fibroblasts (primary)			
Linc-EPS	Multiple	Mouse bone marrow derived macrophages	Represses the inflammatory response by inhibiting IRGs expression	Interacts with hnRNPL via a CANACA motif in its 3′ region and regulates nucleosome positioning at IRG promoters	[86]
LincRNA-Tnfaip3	LPS	Mouse macrophages (RAW 264.7 and primary mouse peritoneal macrophages)	Regulates the expression of several NF-κΒ mediated inflammatory genes	Directly interacts with Hmgb1 and NF-κΒ to form a functional complex to regulate Hmgb1-mediated histone modifications	[87]
Lnc-13	LPS	Human monocytes (primary and U937, THP-1) and mouse bone marrow derived macrophages	Suppresses the expression of several immune-associated genes	Demonstrates NF-κΒ-dependent expression and interacts with Hdac1 on chromatin and hnRNPD to regulate gene expression	[76]
NRIR	LPS	Human monocytes (primary)	Regulates the expression of several interferon-stimulated genes and protein release of CXCL10 and CCL8	Demonstrates type I IFN-dependent expression	[73]
NTT	N/A	Peripheral blood mononuclear cells (PBMCs) Human monocytes (THP-1)	Regulates cell cycle G1 arrest and differentiation as well as expression of IL-10 and CXCL10	Interacts with the TF C/EBPβ and the promoter of its neighbouring gene PBOV1 via hnRNP-U	[88]
Mirt2	LPS	Peritoneal macrophages (C57BL/6 mice) HEK293T and RAW264.7 cells	Regulates macrophage polarisation and aberrant inflammatory activity	Inhibits TRAF6 Lys63-mediated ubiquitination and the activation of the MAPK and NF-κΒ pathways	[75]
Lnc-Lsm3b	Viral RNA molecules	Mouse macrophages (peritoneal, RAW 264.7), L929 and HEK293T cell lines	Inactivates late RIG-1 innate activity and type I IFNs production	Acts as a decoy by saturating RIG-1 binding sites to inhibit inflammation and to prevent tissue host damage	[63]
MALAT1	PMA, LPS	Human monocytes (THP-1), mouse macrophages (RAW 264.7)	Regulates the expression of inflammatory genes such as IL-6 and TNF-α	Interacts with NF-κΒ p50/p65 subunits to inhibit NF-κΒ DNA binding activity	[82,83,93]
	PMA, LPS, IL-4	Mouse macrophages (BMDM), human monocytes (PBMCs, THP-1)	Regulates LPS-mediated M1 macrophage activation and IL-4-mediated M2 differentiation and pro-fibrotic phenotype	Demonstrates *Clec16a*-dependent expression and regulation of mitochondrial pyruvate carriers	
	LPS	Mouse bone-marrow-derived dendritic cells	Induces increased tolerogenic activity of DCs	Enhances DC-SIGN expression, IL-10 production and acts as an miR-155 sponge	
FIRRE	LPS	Human macrophages (U937) Human intestinal epithelial cells (SW480) Mouse macrophages (RAW264.7)	Regulates expression of several inflammatory genes	Demonstrates NF-κΒ-dependent expression and interacts with hnRNPU to regulate mRNA stability by targeting their AREs	[89]
LincRNA-AK170409 LincRNA-Cox2	TLR ligands	Immortalized murine bone marrow–derived macrophages (iBMDM)	Both regulate NF-κΒ-dependent signalling	Both lncRNAs demonstrate NF-κΒ-dependent activity - LincRNA-COX2 promotes IκΒα degradation	[65]
Lnc-DC	N/A	Human conventional dendritic cells	Regulates DC differentiation	Binds directly to STAT3 to prevent dephosphorylation of Y705 by SHP1	[94]
Lethe	TNF-α, IL-1β, dexamethasone	MEF lines (mouse embryonic fibroblasts)	Regulates the expression of several NF-κΒ mediated inflammatory genes	Interacts with the RelA (p65) subunit of NF-κΒ to inhibit DNA binding and gene activation	[96,97]
	Glucose	Mouse macrophages (RAW 264.7 and bone marrow derived macrophages)	Regulates ROS production and NOX2 gene expression	Interacts with the NF-κΒ p65 subunit to control its translocation to the nucleus	
MIR3142HG	IL-1β	Human lung fibroblasts (primary)	Regulates CCL2 and IL-8 mRNA and protein release	Demonstrates NF-κΒ-dependent expression	[60]
NEAT1	Influenza, HSV-1, poly I:C	Human epithelial cells (A549 cell line) and HeLa cells	Regulates expression of IL-8	Interacts and relocates SFPQ from the IL8 promoter to the paraspeckles	[100,101]
	Multiple	HUVEC cells (Human umbilical vein endothelial cells), HEK293 cells, HeLa and 293 T cells	Regulates the DNA-mediated innate immune response	Interacts with HEXIM1 to form the HDP-RNP complex which is required for the cGAS-STING-IRF3 pathway	
LncITPRIP-1	Viral infections	Huh7, Huh7.5, Huh7.5.1-MAVS, FL-neo, and HEK293T cells	Promotes the activation of the innate immune response	Binds to the C-terminus of MDA5 and promotes its oligomerisation to enhance IFN signalling and production	[103]
NEAT1v2 eRNA07573	*Salmonella *infection	HeLa cells	Regulate expression of immune-associated genes and response to antibacterial defence	Inhibit levels of the exosome/NEXT components and demonstrate elevated transcript stability	[102]
